# Interleukin-6 and adhesion molecules VCAM-1 and ICAM-1 as biomarkers of post-acute myocardial infarction heart failure

**DOI:** 10.1590/1414-431X20198658

**Published:** 2019-11-21

**Authors:** D.O.C. Lino, I.A. Freitas, G.C. Meneses, A.M.C. Martins, E.F. Daher, J.H.C. Rocha, G.B. Silva

**Affiliations:** 1 Programa de Pós-Graduação em Saúde Coletiva, Centro de Ciências da Saúde, Universidade de Fortaleza, Fortaleza, CE, Brasil; 2 Serviço de Emergência Cardiológica, Hospital Dr. Carlos Alberto Studart Gomes, Secretaria da Saúde do Estado do Ceará, Fortaleza, CE, Brasil; 3 Departamento de Fisiologia e Farmacologia, Faculdade de Medicina, Universidade Federal do Ceará, Fortaleza, CE, Brasil; 4 Programa de Pós-Graduação em Ciências Médicas, Faculdade de Medicina, Universidade Federal do Ceará, Fortaleza, CE, Brasil

**Keywords:** Heart failure, Acute myocardial infarction, Biomarkers, Prognosis

## Abstract

Acute coronary syndromes are associated with a high prevalence of complications including heart failure (HF). The aim of this study was to investigate the association of novel biomarkers with the occurrence of post-acute myocardial infarction (AMI) HF. A prospective study was conducted with patients admitted to the emergency department with ST-segment elevation myocardial infarction (STEMI). Blood and urine samples were collected for analysis of traditional and novel biomarkers, including interleukin-6, vascular cell adhesion molecule 1 (VCAM-1), and intercellular adhesion molecule-1 (ICAM-1). We compared the levels of these biomarkers between patients with and without post-STEMI HF. A total of 48 patients were assessed, with a prevalence of males. Fifteen patients (31.2%) had post-STEMI HF. Patients with HF had higher mean values of IL-6, VCAM-1, and ICAM-1 compared to those who did not develop HF (57.06 *vs* 14.03 pg/mL, P=0.001; 1719.58 *vs* 1304.34 ng/mL, P=0.001; and 1594.20 *vs* 1158.74 ng/mL, P<0.001, respectively). The three biomarkers were shown to be good predictors of post-STEMI HF (IL-6: AUC 0.786, P=0.002; VCAM-1: AUC 0.797, P=0.001; and ICAM-1: AUC 0.825, P<0.0001), with the respective cutoff points being calculated based on the best sensitivity and specificity indexes (IL-6: 8.67 pg/mL; VCAM-1: 1501.42 ng/mL; and ICAM-1: 1262.38 ng/mL). Of the three biomarkers, only VCAM-1 and ICAM-1 had a direct linear association between them (r=0.470, P<0.0001). IL-6, VCAM-1, and ICAM-1 were associated with the development of new post-AMI HF symptoms, but only VCAM-1 and ICAM-1 correlated with each other, possibly because they have the same pathophysiological mechanism of action.

## Introduction

In general, all acute coronary syndromes (ACS), including unstable angina, ST-elevation acute myocardial infarction (STEMI), and non-ST elevation myocardial infarction are associated with cardiomyocyte loss, fibrosis, and cardiac remodeling, which together represent the main pathophysiological mechanisms that comprise the clinical picture of heart failure (HF) and are associated with high morbidity and mortality worldwide (1).

Several prognostic scores have been used as complication predictors in acute myocardial infarction (AMI), such as the TIMI Risk, GRACE, PREDICT, and the PURSUIT scores, but they only define the chance of the total risk of death (or reinfarction), not considering specific secondary events, such as the onset of HF. However, HF is much more frequent and represents an important additional cost to health systems. According to the Heart Diseases and Stroke Statistics 2018, 17 to 21% of individuals over 45 years of age who had had the first AMI had HF after a 5-year follow-up (2–7).

The National Institutes of Health define a biological marker or biomarker as a test that is an objective measure, which indicates normal biological or pathogenic processes and responses to therapy. This could be a blood test, an imaging study, or a hemodynamic value (7). Biomarkers that evaluate cardiovascular function may encompass a wide range of biochemical or physiological measurements. The clinical usefulness of a biomarker is context-dependent. Regarding ACS, some biomarkers have been investigated, including interleukins and adhesion molecules (VCAM-1 and ICAM-1), which are involved in the process of atherosclerosis and inflammation. Regarding HF, biomarkers are used as diagnostic and prognostic tools, as well as in the monitoring of acute cell rejection in heart transplant recipients. Other possibilities may include early detection in individuals with suspected HF, risk assessment in subjects without HF, and selection of patients who will benefit from a specific therapy (8–11).

Considering HF a significant clinical complication within the AMI context, the aim of this study was to investigate the association of the biomarkers interleukin-6 (IL-6), vascular cell adhesion molecule 1 (VCAM-1), and intercellular adhesion molecule 1 (ICAM-1) with the development of post-AMI HF during a hospital stay due to the AMI.

## Material and Methods

### Patient selection

A prospective study was carried out including patients admitted with ST-segment elevation myocardial infarction (STEMI) at the Emergency Department of Dr. Carlos Alberto Studart Gomes Hospital, in the city of Fortaleza, Northeastern Brazil, which is a referral hospital in Cardiology for the region. The study protocol was reviewed and approved by the Hospital's Research Ethics Committee, according to the ethical guidelines of the 1975 Declaration of Helsinki (protocol number: 66658017.4.0000.5039).

The study included patients admitted to the Emergency Department from March to July 2018 with a diagnosis of STEMI, aged 18 years or older, who signed the free and informed consent form. Patients with neoplasms, ongoing infections, chronic kidney disease undergoing dialysis, pregnant women, whose diagnosis might have been modified during hospitalization, or those who left before the main exams were performed, and patients who did not meet the criteria for the presence/absence of outcomes were excluded.

### Study protocol

The initial phase of the study consisted of an interview, during which a questionnaire was applied, containing socioeconomic information, personal and family medical history, history of previous cardiovascular events, and alcohol and tobacco consumption. A physical examination was performed, and blood pressure and glycemia levels were recorded at admission. Data were collected on the infarction location, between symptom onset, and the reperfusion strategy (chemical or mechanical). After the initial analysis, the patients were followed clinically during their hospital stay until discharge or death. The presence of new symptoms of HF were recorded based on the Boston criteria (2).

### Laboratory tests

After the initial interview, blood and urine samples were concomitantly collected for analysis, and the samples were stored and refrigerated at −80°C until the analysis was performed. The following analyses were carried out: serum levels of glucose, creatinine, urea, sodium, potassium, chloride, magnesium, total cholesterol, low-density lipoprotein (LDL), high-density lipoprotein (HDL), triglycerides, and urinalysis, including proteinuria. Glomerular filtration rate (GFR) was estimated using the CKD-EPI equation (12). The biomarkers IL-6 (Human IL-6 DuoSet ELISA DY206-05: R&D Systems, USA) and adhesion molecules VCAM-1 and ICAM-1 (Human ICAM-1 ELISA Set (without plates) (ab47349) and Human VCAM-1 ELISA Set (without plates) (ab47355) Abcam, USA) were also measured (13,14).

### Statistical analysis

Statistical analysis was performed using the SPSS program, version 20.0 (IBM, USA). Comparison of parameters between the two groups (presence or absence of HF) was performed using the Mann-Whitney test and Fisher's exact test. The evaluation of IL-6, VCAM-1, and ICAM-1 as predictive factors for new HF post-AMI was performed using predicted probability curves and diagnostic accuracy assessment using receiver operating characteristics (ROC) analysis. Non-adjusted odds ratios (ORs) and 95% confidence intervals (CI) were calculated. Spearman's correlation coefficient was used to assess the linear correlations between the biomarkers. Significance was set at P<0.05.

## Results

A total of 48 patients were assessed, with a mean age of 61.4±10.4 years, and a prevalence of males (66.7% in the group of patients with HF and 78.8% in the group without HF). There was no significant difference in relation to the medical history prior to admission regarding smoking, presence of diabetes, and arterial hypertension. Regarding the general laboratory tests, there was no statistical difference between the means of leukocyte counts (15,266±4,636 *vs* 11,769±3,843; P=0.01) and levels of C-reactive protein were higher in the group with HF (7.97±7.86 *vs* 1.48 ± 1.74, P=0.002). Regarding imaging tests, the group of patients with HF had a higher mean syntax score (21.39±10.07 *vs* 12.85±7.46) and lower ejection fraction (43.47 *vs* 51.09%). The IL-6, VCAM-1, and ICAM-1 means were significantly higher in the HF group.

**Figure 1.**
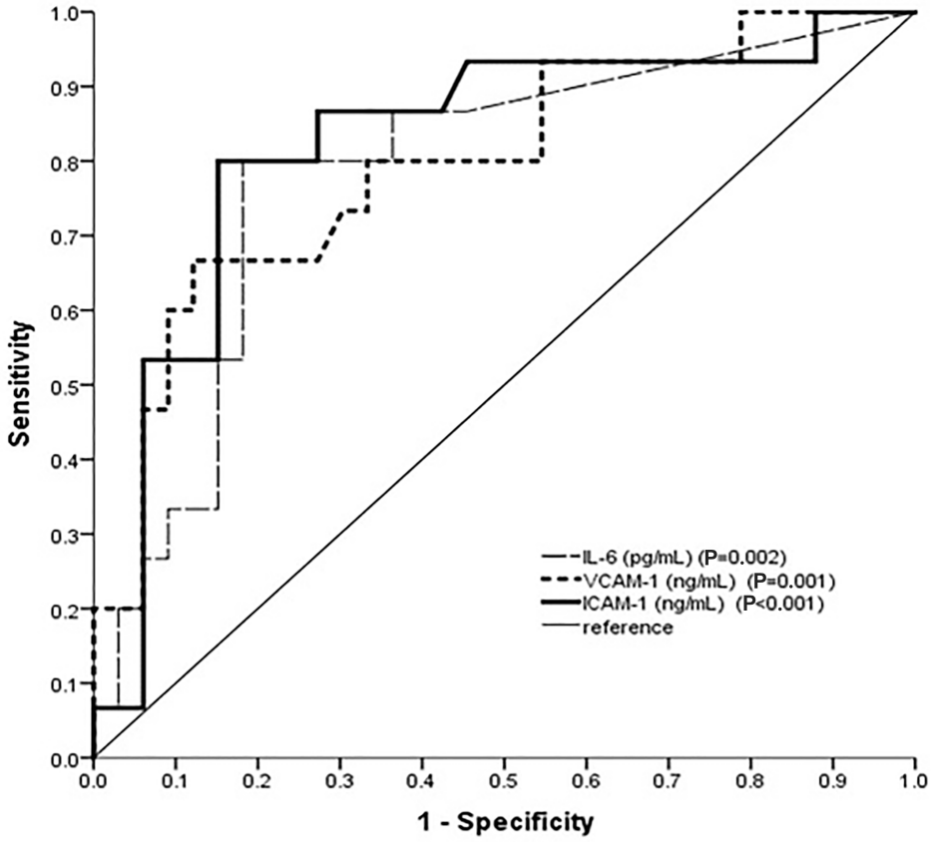
Receiver operating characteristics of interleukin-6 (IL-6), vascular cell adhesion molecule 1 (VCAM-1), and intercellular adhesion molecule 1 (ICAM-1) as biomarkers of post-acute myocardial infarction heart failure.

Figure 1 shows a chart with the predicted probabilities for new HF post-AMI against serum levels of IL-6, VCAM-1, and ICAM-1 at admission. The areas under the ROC curve (AUC) were 0.786 (P=0.002), 0.797 (P=0.001), and 0.825, (P<0.0001) respectively. A cutoff point of 8.676 pg/mL was selected for IL-6 to optimize sensitivity and specificity. The same analysis was performed for VCAM-1 and ICAM-1, with the respective cutoff points of 1501.42 ng/mL and 1262.38 ng/mL. The positive predictive values of IL-6, VCAM-1, and ICAM-1 were 0.66, 0.52, and 0.59 and the negative predictive values were 0.90, 0.88, and 0.92, with their respective accuracy values of 81.25, 70.83, and 77.08%. The number of patients with new HF post-AMI was significantly higher in the groups that had higher biomarker values, according to the established cutoff points (Table 1). Only VCAM-1 and ICAM-1 had a significant positive association between them (r=0.470, P<0.0001) (Table 2).

**Table 1.** Clinical and laboratory characteristics of the patients according to the presence or absence of post-acute myocardial infarction new heart failure (HF).

	Presence of HF (n=15)	Absence of HF (n=33)	P value
Age (years)	62.47±12.65	60.94±9.39	0.578
Gender			
Male	10 (66.7%)	26 (78. %)	0.476
Female	5 (33.3%)	7 (21.2%)	
Smoking	10 (66.7%)	17 (51.5%)	0.366
Diabetes	7 (46.7%)	8 (24.2%)	0.18
Arterial hypertension	6 (40%)	19 (57.6%)	0.353
Location of AMI			
Anterior	11 (73.3%)	7 (21.9%)	0.001
Inferior	4 (26.7%)	24 (75%)	0.003
Hemoglobin (mg/dL)	13.09±2.3	13.85±1.66	0.209
Leukocytes	15266±4.636	11769±3.843	0.01
Creatinine (mg/dL)	1.23 ±0.55	1.04±0.31	0.373
CRP	7.97±7.86	1.48±1.74	0.002
Troponin	8.36±3.88	5.51±3.59	0.024
Syntax score	21.39±10.07	12.85±7.46	0.006
Ejection fraction (%)	43.47%	51.09%	0.007
IL-6 (pg/mL)	57.06±111.08	14.03±39.71	0.001
VCAM-1 (ng/mL)	1719.58±338.94	1304.34±329.79	0.001
ICAM-1 (ng/mL)	1594.20±363.20	1158.74±339.67	<0.001
Medication			
Aspirin	15 (100%)	33 (100%)	-
P2Y12 inhibitor	15 (100%)	33 (100%)	-
Statin	14 (93.3%)	28 (90.3%)	1.00
Enoxaparin	9 (60%)	14 (45.2%)	0.53
β-blocker	8 (53.3%)	15 (48.4%)	1.00
ACEi/ARBs	8 (53.3%)	21 (67.7 %)	0.516

Data are reported as mean±SD or number and percentage. Mann-Whitney test and Fisher's exact test were used for statistical analysis. AMI: acute myocardial infarction; CRP: C-reactive protein; IL-6: interleukin-6; VCAM-1: vascular cell adhesion molecule 1; ICAM-1: intercellular adhesion molecule-1; ACEi: angiotensin-converting enzyme inhibitors; ARBs: angiotensin II receptor blockers.

**Table 2.** Association between established cutoff points for IL-6, VCAM-1, and ICAM-1 with the presence of new heart failure post-acute myocardial infarction (HF).

Biomarker	Presence of HF		Absence of HF		P	OR	95% CI
	N	%	N	%			
IL-6 (pg/mL)					<0.0001		
≥8,676	12	66.7%	6	33.3%		18.0	3.844-84.277
<8,676	3	10.0%	27	90.0%		1.0	
VCAM-1 (ng/mL)					0.004		
≥1501.429	12	52.2%	11	47.8%		8.0	1.862-34.363
<1501.429	3	12.0%	22	88.0%		1.0	
ICAM-1 (ng/mL)					<0.0001		
≥1262.387	13	59.1%	9	40.9%		17.33	3.240-92.470
<1262.387	2	7.7%	24	94.3%		1.0	

IL-6: interleukin-6; VCAM-1: vascular cell adhesion molecule 1; ICAM-1: intercellular adhesion molecule -1; CRP: C-reactive protein; ACEi: angiotensin-converting enzyme inhibitors; ARBs: angiotensin II receptor blockers. Spearman's correlation coefficient was used for statistical analysis.

## Discussion

The results obtained in this study showed that inflammatory markers, which can be detected using relatively simple and easily accessible laboratory techniques, may be associated with the prognosis of patients with AMI, regarding HF development. Plasma levels of IL-6, VCAM-1, and ICAM-1 were high in patients who developed post-AMI HF. By using the ROC curves and defining a cutoff with better accuracy, IL-6, VCAM-1, and ICAM-1 showed a significant contribution in predicting the occurrence of HF after the ischemic event, with ICAM-1 having the best predictive ability. After performing a correlation analysis between the biomarkers, a direct positive linear association between VCAM-1 and ICAM-1 was observed, which did not occur between these markers and IL-6, a fact not yet reported in the scientific literature in this clinical context.

HF is the terminal outcome and the end-stage of many cardiovascular diseases, and it severely impacts the quality of life of affected patients. A growing body of evidence has shown that increased plasma levels of proinflammatory cytokines and other biomarkers have prognostic implications in HF. It is also noteworthy that new-onset HF has been analyzed in a few studies, while it has often been incorporated into a composite endpoint including re-infarction, HF, or death. However, it is clear that this approach does not truly mirror the clinical reality in the AMI context, given that the number of individuals who develop new HF onset after AMI is much higher than those who experience re-infarction or death (1).

IL-6 is a 26-kDa protein produced by the liver and considered to be crucial in the acute-phase inflammatory response, promoting lymphocyte activation and proliferation, B-cell differentiation, leukocyte recruitment, and regulation of the synthesis of acute phase proteins, fibrinogen, and albumin (13). Among cytokines, IL-6 has been suggested as the best marker of disease severity, considering the increased plasma concentrations and activated myocardial gene expression. It has been demonstrated that IL-6 circulating levels are associated with increased morbidity and mortality in acute and chronic myocardial infarction. In the HF context, IL-6 was significantly correlated with plasma norepinephrine concentrations in patients with chronic HF and inversely correlated with functional status in left ventricular dysfunction studies (15,16).

Many studies have shown that IL-6 participates in the pathophysiology of AMI and cardiac remodeling. However, other studies have reported that carriers of IL-6 gene knockdown showed no influence on infarct size, ventricular remodeling, left ventricular function, or mortality at prolonged follow-up after AMI induction in experimental animals. Perhaps in terms of long-term remodeling, IL-6 does not have a major contribution to ventricular adaptation (15).

Few studies in the literature have performed the specific analysis of IL-6 and assessed the outcome of post-AMI HF, mainly in the index event. Lippi and Cervellin (1), in a systematic review that focused on the risk assessment of developing post-AMI new congestive heart failure (CHF), identified 447 articles, in which 3 patients were selected from a sample of over 100 patients, indicating the scarcity of robust studies on the subject.

Studies with human subjects have shown differences regarding results. Kavsak et al. (17), in a study of 216 patients, reported that high IL-6 levels were a significant predictor of HF in the first 6 months, 2 years, and 8 years post-event (6 months: HR 3.79, 95%CI: 1.28–11.19, P=0.016; 2 years: HR 3.27, 95%CI: 1.48–7.20, P=0.003; and 8 years: HR 2.57, 95%CI: 1.58–4.19, P=0.001). Hartford et al. (18), in an analysis of 134 patients with AMI after a 30-month follow-up, did not find any significant differences in IL-6 levels between patients who were hospitalized for HF and those who were not (18.9 *vs* 17.5 pg/mL, P=0.39).

Vascular cell adhesion protein-1, also known as vascular cell adhesion molecule-1 (VCAM-1) or cluster of differentiation 106 (CD 106), is a protein encoded by the *VCAM-1* gene in humans. VCAM-1 functions as a cell adhesion molecule, mediating the adhesion of lymphocytes, monocytes, eosinophils, and basophils to the vascular endothelium and may play a role in the development of atherosclerosis (19).

ICAM-1, also known as cluster of differentiation 54 (CD54), is a cell surface glycoprotein typically expressed in endothelial and immune system cells, and its association with immune responses indicated it acts as a signal transducer. Its binding to integrins produces proinflammatory effects, such as leukocyte recruitment. However, few studies have evaluated its effects in HF, especially in ischemic patients. The latest update by the American Heart Association on biomarkers and HF does not even mention the adhesion molecules in this group of patients (20,21).

Regarding ACS, a study that included 75 patients measured circulating levels of ICAM-1 and VCAM-1, VCAM-1 showed to be a powerful predictor of major events in this patient profile; however, a new coronary event, hospitalization for angina, or cardiac death were considered adverse outcomes. According to Postadzhiyan et al. (22), many assertions support the hypothesis that cell adhesion molecules may be more useful markers than the other inflammatory markers in predicting events in patients with ACS.

In a subgroup of the PRIME study that evaluated a total of 9,758 healthy patients and 317 patients who suffered an acute event, serum levels of ICAM-1 and VCAM-1 were measured. The patients were followed for 5 years regarding the development of adverse events (including death, angina, and AMI). Elevated plasma levels of ICAM-1 were associated with the risk of AMI, coronary death, and angina during follow-up, an outcome not observed when serum levels of VCAM-1 were assessed (20).

Lemos et al. (23), in a study (Physicians' Health Study, PHS) similar to PRIME, evaluated serum levels of VCAM-1 and ICAM-1, which were compared to controls (474 patients in each group) regarding future coronary events (again not including aspects related to HF as the outcome).

Concurrent results, similar to another multicenter study, the ARIC (Atherosclerosis Risk in Communities) study, showed that high levels of ICAM-1, but not of VCAM-1, were significantly associated with AMI prediction in healthy individuals, leading to the assumption that, in this context, there are differences between VCAM-1 and ICAM-1 levels in atherothrombosis genesis (23).

Regarding HF, Savic-Radojevic et al. (24), in a study of 120 patients with chronic HF and 69 controls, found that both ICAM-1 and VCAM-1 levels did not differ significantly in patients at early clinical stages in relation to controls. However, elevated levels of VCAM-1 were found in NYHA class III/IV patients, compared to those with NYHA class I/II. As for ICAM-1, its levels are slightly elevated in NYHA class I/II patients and increase significantly in patients with NYHA class III/IV (more severe stages of HF). It should be highlighted that this analysis was performed in patients with chronic disease presentation, and there was no report in the literature within a context of a new HF after a coronary event (24).

The results of our study should be considered according to its limitations. It was a small study, with a small number of patients. Moreover, these results should be used as the basis for larger prospective studies. Because of the heterogeneity of available results, the evaluation through panels of biomarkers with different pathophysiological mechanisms may be more cost-effective in identifying those patients at higher risk for developing HF and who can benefit from early clinical and therapeutic management strategies.
